# Screening for Peptides to Bind and Functionally Inhibit SARS-CoV-2 Fusion Peptide Using Mirrored Combinatorial Phage Display and Human Proteomic Phage Display

**DOI:** 10.3390/molecules31020282

**Published:** 2026-01-13

**Authors:** Ajay Pal, Neeladri Sekhar Roy, Matthew Angeliadis, Priyanka Madhu, Sophie O’Reilly, Indrani Bera, Nathan Francois, Aisling Lynch, Virginie Gautier, Marc Devocelle, David J. O’Connell, Denis C. Shields

**Affiliations:** 1 School of Medicine, University College Dublin, D04 C1P1 Dublin, Irelandindrani.bera@ucd.ie (I.B.);; 2 Conway Institute of Biomolecular and Biomedical Research, University College Dublin, D04 C1P1 Dublin, Ireland; david.oconnell@ucd.ie; 3 Department of Chemistry, Royal College of Surgeons in Ireland, D02 YN77 Dublin, Irelandmdevocelle@rcsi.ie (M.D.); 4 Centre for Experimental Pathogen and Host Response, University College Dublin, D04 V1W8 Dublin, Ireland; 5 Department of Chemistry, Uppsala University, 751 05 Uppsala, Sweden; 6 The Group of Applied Physics, School of Physics, Clinical and Optometric Sciences, Technological University Dublin, D07 ADY7 Dublin, Ireland; 7 School of Biomolecular and Biomedical Sciences, University College Dublin, D04 V1W8 Dublin, Ireland

**Keywords:** SARS-CoV-2, fusion peptide, combinatorial peptide phage display, proteomics peptide phage display, SARS-CoV-2 inhibitors, retro-inverso peptides, OTUD1

## Abstract

To identify pancoronaviral inhibitors, we sought to identify peptides that bound the evolutionarily conserved SARS-CoV-2 spike fusion peptide (FP). We screened the NEB PhD-7-mer random combinatorial phage display library against FP, synthesised as a D-peptide, to identify peptides from the L-library to be synthesised as proteolytically resistant D peptides. We selected the top ten peptides that were not seen in another published screen with this library, as these were more likely to be specific. All ten D-peptides had no impact on the infection of Vero-E6/TMPRSS2 cells by SARS-CoV-2. Screening of a proteomic-derived phage display library from the disordered regions of human proteins identified two overlapping 14mer peptides from a region of OTUD1. While a synthetic peptide based on their sequences failed to markedly inhibit viral entry, molecular dynamics structural modelling highlighted a stable binding mode where positive residues on one side of the OTUD1 helix interacted with hydrophobic residues of the FP triple-helical wedge. Thus, while the two phage display strategies failed to yield peptide sequences that are themselves strong inhibitors of viral infection, they led to the development of a computational model that can underpin future designs of potential pancoronaviral FP disruptors.

## 1. Introduction

The development of a diverse range of antiviral therapies against SARS-CoV-2 and other coronaviruses is essential in preparing for the burden of future coronaviral pandemics and epidemics on public health [1]. Surface proteins on viral membranes are key mediators of viral infection in host cells. Synthetic peptides derived from the fusion peptide (FP) and heptad repeat (HR) regions of measles virus and human immunodeficiency virus type-I (HIV-1) have been reported to inhibit viral replication, thus being used as antiviral peptides in subacute sclerosing panencephalitis [2] and HIV-1 infection [3]. Coronaviruses are similarly enveloped viruses with an exterior lipid envelope. Their major surface glycoprotein, Spike (S), contains FP and HR regions that play key roles in the fusion of viral and host membranes to transfer the viral genetic material into the host cell [4]. Upon binding of the receptor binding domain of S to the major host receptor protein ACE2, S undergoes a conformational change involving HR multimerization that exposes FP and promotes FP insertion into the host cell membrane [4], typically following the FP N-terminus being exposed during S activation by TMPRSS2 proteolytic cleavage between the S1 and S2 regions of S. Peptides of around forty amino acids in length derived from the HR region have been shown to inhibit SARS and SARS-CoV-2 infection in animal models [5]. The role of FP-derived or FP-targeting peptides in inhibiting coronaviral infection has not been extensively explored. Given FP evolutionary conservation across coronaviruses, any viral entry inhibitors targeting it may be less prone to the evolution of viral resistance and may be useful in inhibiting multiple coronaviral species.

FP has been defined as encompassing two regions: the N-terminal FP1 and the following FP2 region. The structure of FP has been characterised in a membrane bicelle as a wedge-shaped set of three alpha-helices. This structure enables interactions of its exposed hydrophobic residues with phospholipid acyl chains [6], with a short disulphide bridge in the FP2 region contributing in part to the structure. Phosphorylation of the serine at the very start of FP has been reported [7], but it is unclear what proportion of S proteins are phosphorylated at this site, either before or after protease cleavage upstream of FP. Addition of this negatively charged post-translational modification may not directly disrupt the wedge-like FP structure, since the serine is exposed, but it could alter S and FP interactions with the membrane, interacting proteins or inhibitors.

We set out to identify peptides that bind the non-phosphorylated form of FP and that might also have the potential to disrupt SARS-CoV-2 viral entry. To identify binding peptides, we screened two phage display libraries. The NEB PhD-7-mer library is a diverse library of a billion (10^9^) random 7-mer peptides displayed on the surface of M13 bacteriophage. Since peptides containing D-amino acid enantiomers are more resistant to proteolytic degradation, we identified D-amino acid binders of FP using a mirror phage display approach [8]. While retro-inverso (D)-peptides only retain an approximate functionality of forward L-peptides, with mirrored phage display there is a nicely preserved perfect fit of the selected peptides, since the screened D-FP bait has an interaction with the phage display binding L-peptides that is an exact mirror image of the L-peptide FP (natural) sequence interaction with the phage display peptides sequenced as D-amino acids [8]. Secondly, we screened a proteomic phage display library derived from the disordered regions of human proteins [9], since it is possible that FP may form interactions with human protein regions during membrane fusion. Peptides of interest derived from both screening strategies were then tested for their ability to inhibit viral entry, and a computational model of FP-peptide binding was then developed for the peptide of greatest interest.

## 2. Results

### 2.1. Binding Kinetics Between FP and Selected S-Derived Peptides

We initially hypothesised that S-derived peptides that affect viral entry could potentially interact with FP. A number of regions of S were identified on the basis of their potential for FP interaction after S re-organises following fusion (mainly membrane embedding potential) and/or the ability of known homologous peptides from SARS-CoV to inhibit viral entry [10]. We evaluated the synthesised SARS-CoV-2 peptide binding kinetics with cyclic-FP-Biotin attached to a streptavidin-coated Surface Plasmon Resonance (SPR) surface chip.

The peptide sequence was based on amino acids 816–855 spanning the overlapping fusion peptide 1 and 2 regions of UNIPROT spike protein entry P0DTC2. The peptide sequence was SFIEDLLFNKVTLADAGFIKQYGDCLGDIAARDLICAQKF-GGK(biotin)-am, where GGK provides an additional linker, with the K providing an attachment site for biotin, and “-am” indicates amidation of the carboxy terminus; the two cysteines are disulphide bonded. We chose to use the cyclic form of the fusion peptide as bait. This is motivated by the strong conservation of the SARS fusion peptide disulphide bond, whose functional importance is indicated both from structural proximity of the two cysteines and from mutational analyses [11]. This role is also supported by computational modelling in SARS-CoV-2 showing that the disulphide bond can stabilise alpha helical conformations and promote membrane interactions [12,13]. Mass spectrometry of the synthesised bait confirmed that it was in the disulfide-bonded form.

The amino terminus is the free amino terminus that is exposed on the Fusion peptide following protease cleavage during activation, typically by the host TMPRSS2 protease. None of these candidate selected peptides (Table 1) bound to FP as a negligible response signal was recorded for each of the four peptides (Supplementary Figure S2). In the absence of relevant positive controls for binding to the full FP, we inspected the SPR traces, which indicated that the cyclic-FP-biotin had been immobilised successfully, before we went on to assess peptide binding. While we cannot rule out binding at much higher concentrations, these would be harder to detect versus background noise, and of much less biological interest to follow up.

### 2.2. Identification and Testing of Combinatorial Phage Display Peptides

**Figure 1:**
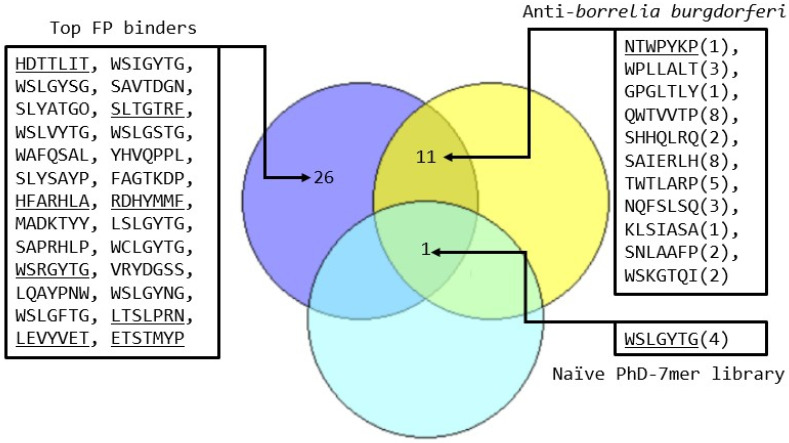
Venn diagram of most frequent FP binding peptides (blue) compared with those returned in an extensively reported NEB PhD7 screened dataset against anti-*Borrelia burgdorferi* immune sera [4] (yellow) and with a naïve library [6] (cyan). Underlined peptides represent those selected for the antiviral assay. Numbers in parentheses denote the occurrences of the FP binding peptide in the anti-*Borrelia burgdorferi* immune sera screening dataset.

We screened a combinatorial phage display library of 7-mer peptides for binding to the FP. The genome sequencing of 177 bp (PhD7-mer NEB) PCR products resulted in 7,143,870 sequence reads (2,143,161,000 sequenced bases). Parsing of the sequence reads using in-house Python scripts yielded 15,313 unique peptide sequences out of which only 80 unique peptides had an abundance greater than 2550. The peptide sequence WSLGYTG was found to be the most enriched with a sequence count of 6,280,489. Recent work has shown that the enrichment of the peptide sequence “WSLGYTG” is target-unrelated, with its enrichment due to its propagation advantage in the M13KE-based peptide library [17]. To filter out sequences that had such an unfair propagation advantage, we filtered out matches to two sources of enriched peptides (Figure 1) that were not specific for the FP target: a naïve amplified PhD7-mer NEB library [18] and a screen of the same library against a target that had extensive reporting of detected sequences [19].

The naïve library only shared the WSLGYTG artefact. A greater number of matching peptides were observed (Figure 1) among PhD7-mer peptides binding to anti-*Borrelia burgdorferi* immune sera [19], and were accordingly removed from our candidate list, as they are less likely to be target-specific. The final selection of peptide sequences for antiviral assays (Table 2) excluded certain peptides where there were concerns about the stability or the synthetic feasibility of the peptides [20].

### 2.3. Screening Versus the Human Disordered Proteomic Phage Display Library

A proteomic phage library of human disordered protein regions [9,21] was screened against spike-FP12 peptide, in duplicate (Table 3). Some of the identified peptides (CFAP73, CFAP65, MAP1A) are known to be over-enriched in a target non-specific manner in screens with this library [21] and were thus considered lower priority.

Two retrieved peptides highlighted a stretch of ovarian tumour domain-containing protein 1 (OTU deubiquitinase 1, OTUD1). These overlapping peptides shared the sequence NFRLSEHRQALA.

Proteomic phage display of peptide regions of proteins is also possible for viral as well as host proteins, and a preliminary screen of FP versus a pan-coronaviral phage display library of regions of coronaviral proteins was carried out following the methods previously outlined [22], which only identified a single peptide, despite a large amount of sequencing data obtained. This 39mer LSTLSVDFNGVLHKAYIDVLRNSFGKDLNANMSLAECKR peptide was derived from the nsp3 (protease-encoding) protein of coronavirus 229E comprising the last four residues of the loop between Y2 and Y3 regions and the first 35 residues of the Y3 region. Y3 plays a role in double-membrane vesicle formation [23], but the lack of binding to homologous regions of other coronaviruses in the library placed the finding in doubt.

### 2.4. Structural Modelling of Interactions Between OTUD1 Peptide and FP

**Figure 2:**
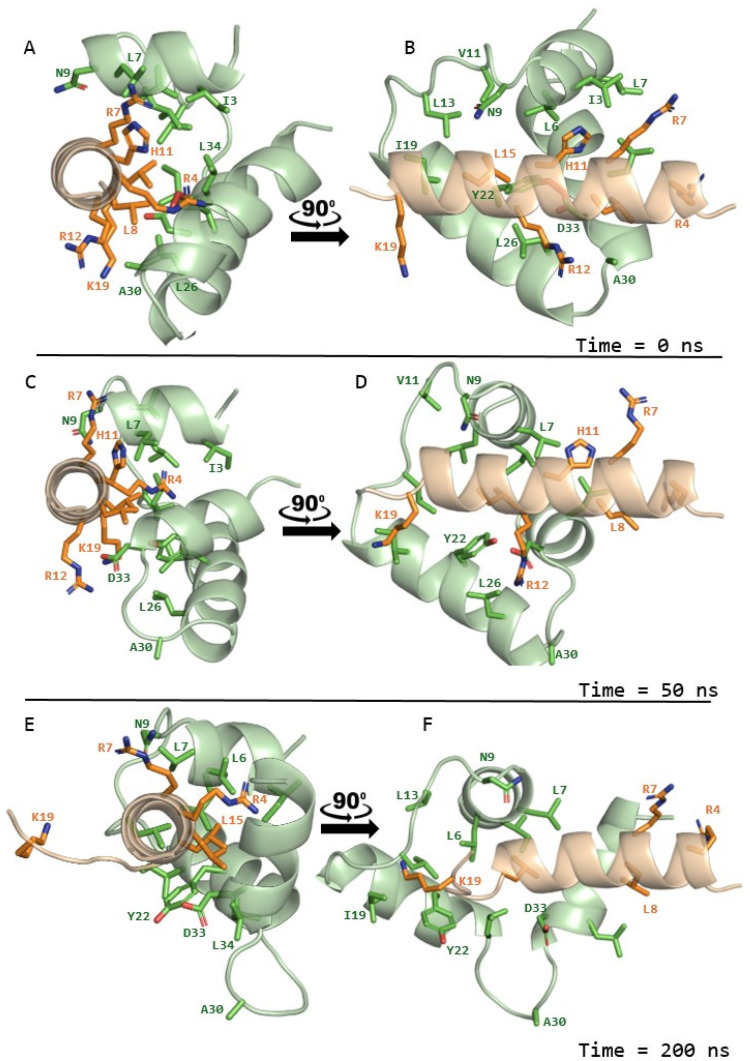
(**A**) Cartoon representation of the overall predicted model of OTUD1 (in green) peptide bound to FP (in cyan) from top, Oxygen and Nitrogen atoms are highlighted in red and blue colour respectively. (**B**) Side view of bound pose with H-bond interactions (in yellow) of OTUD1 with predominantly positively charged FP adjacent residues in magenta (R4, R7, L8, H11, R12, L15, K19) and OTUD1-adjacent predominantly hydrophobic residues of FP shown in orange (I3, L6, L7, N9, V11, L13, I19, Y22, L26, A30, D33, L34). (**C**) MD simulation after 50 ns, top view (**D**) MD simulation after 50 ns, side view (**E**) MD simulation after 200 ns, top view (**F**) MD simulation after 200 ns, side view.

We then investigated structural predictions of OTUD1 peptide binding modes to FP. We predicted the structural complex of the OTUD1 region spanned by both peptides to FP, using the Alphafold3 [24] server. Alphafold3 drew on a template library (up to the cutoff of 30 September 2021) that included the wedge-shaped triple helix conformation of FP from an NMR model in membrane bicelles (PDB entry 7MY8; [6]). The suggested binding mode of OTUD1 to FP did involve a three-helix, approximately wedge-like FP structure, in which the FP residues that interacted with OTUD1 were predominantly hydrophobic, including three of the phospholipid acyl binding residues identified in the bicelle structure, while five of the seven residues of the helical OTUD1 peptide at the interaction surface were positively charged (Figure 2).

We sought to determine whether this predicted structure was likely to be stable. The molecular dynamics (MD) simulations show that OTUD1 remained in complex with FP throughout the 200 ns trajectory. Both the total energy and potential energy of the system dropped sharply during the initial phase of the simulation and stabilised after about 40–60 ns, confirming that the system had reached equilibrium and remained energetically stable for the rest of the run (Supplementary Figure S3a,b). The root-mean-square deviation (RMSD) analysis revealed that OTUD1 maintained a steady backbone structure with only minor fluctuations after equilibration, while the FP exhibited slightly higher mobility. This behaviour suggests that OTUD1 stayed structurally rigid, whereas FP adjusted its conformation before settling into a stable pose (Supplementary Figure S3c), which can be seen in a movie, showing the flip of the last alpha-helix of FP from the initial location to an alternative location, where it appeared stable, without flipping back again (see DOI 10.5281/zenodo.17898744. Most residues in OTUD1 displayed very low flexibility, with higher fluctuations at the ends of the FP, consistent with the natural flexibility of terminal regions involved in binding (Supplementary Figure S3d).

Contact and minimum-distance analyses between OTUD1 and FP revealed that several of the OTUD1 residues originally predicted to lie at the interface—namely, R4, R7, L8, H11, R12, L15, and K19—maintained persistent interactions with FP over the course of the MD run. For the majority of frames (>~70%), these residues remained within the contact cutoff distance (≤0.6 nm) (Supplementary Figure S3e), indicating a stable and specific interface. Hydrogen-bond occupancy analysis further supported that a subset of these contacts involved stable hydrogen bonds (Supplementary Figure S3f), suggesting that the OTUD1–FP association predicted by AlphaFold3 is dynamically stable under explicit-solvent MD conditions.

To characterise this further, we selected the 50 ns and 200 ns models as representatives of the overall trajectory of simulation. The complex structures (Figure 2) showed overall similarity, with some differences between these two models. LIGPLOT [25] analyses identified the key interacting residues that were constant across both these models, namely, that FP Leu 6 and Leu 7 interacted hydrophobically with OTUD1 residues 12 to 18, FP Leu 34 had hydrophobic interaction with OTUD1 Leu8 and His11, and a salt bridge between FP Asp33 and OTUD1 Arg12 (Supplementary Figure S4).

These results demonstrate that the OTUD1–FP complex initially predicted by AlphaFold3 appears both structurally and energetically stable under physiological conditions, with the third alpha helix of the FP showing two alternative orientations. In vivo, the stabilisation provided by the rest of the S2’ region may well impact on which of these two states might be most occupied.

### 2.5. Inhibition of SARS-CoV-2 Infection and Toxicity

**Figure 3:**
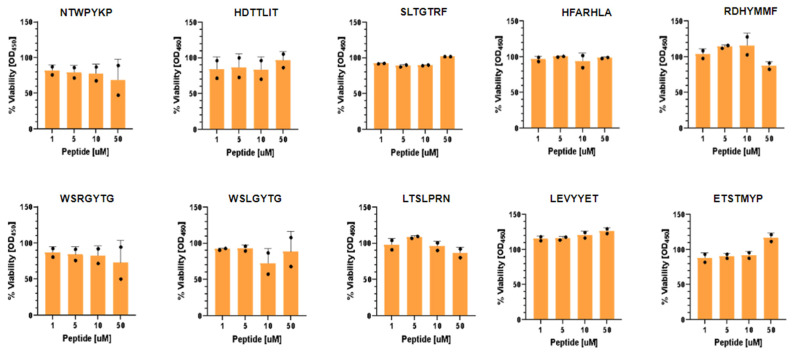
Cell viability assays of Vero-E6/TMPRSS2 cells in the presence of peptides selected from the PhD7 combinatorial library. Black dots represent the data points.

We assessed the toxicity and efficacy of the ten selected 7-mer D peptides in Vero-E6/TMPRSS2 cells. Vero E6/TMPRSS2 cells are an African Green Monkey kidney epithelial cell line that endogenously expresses the ACE2 receptor [26,27] and are modified to constitutively express the TMPRSS2 receptor, rendering them highly permissive to SARS-CoV-2 infection. We first evaluated their cytotoxicity using a CCK8 viability assay. Cells were treated for 18 h with peptide concentrations ranging from 1 to 50 μM. No significant toxicity was observed with viability remaining between 70% and 100% across all tested conditions (Figure 3).

**Figure 4:**
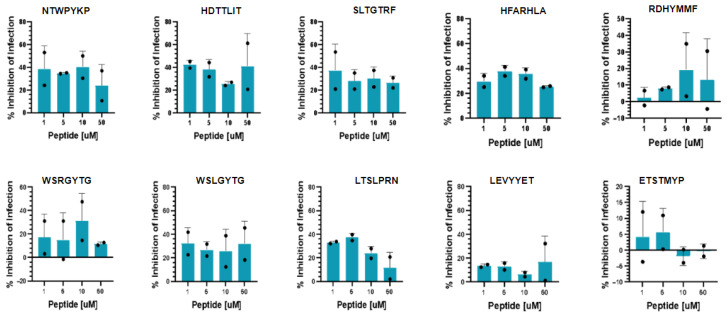
Inhibition of SARS-CoV-2 infection of Vero-E6/TMPRSS2 cells by selected peptides (synthesised as D peptides) from the PhD7 combinatorial library. Data is normalised against vehicle-treated infected cells. Plots show the mean and standard deviation of 2 independent experiments.

We next examined the antiviral capacity of the combinatorial peptides against SARS-CoV-2 infection in vitro. Vero-E6/TMPRSS2 were pre-incubated with the peptides (1–50 μM) for 2 h prior to infection with SARS-CoV-2. The infection of these cells was strongly inhibited by the known anti-coronaviral Remdesivir (Supplementary Figure S5). After 18 h of infection in the presence of the peptides, we observed a moderate reduction in the number of infected cells in the peptide-treated wells compared to the vehicle controls with 9/10 peptides with the exception of ETSTMYP. However, no dose–response was observed, and the maximum inhibition remained modest, achieving less than 50% reduction in infection (Figure 4).

**Figure 5:**
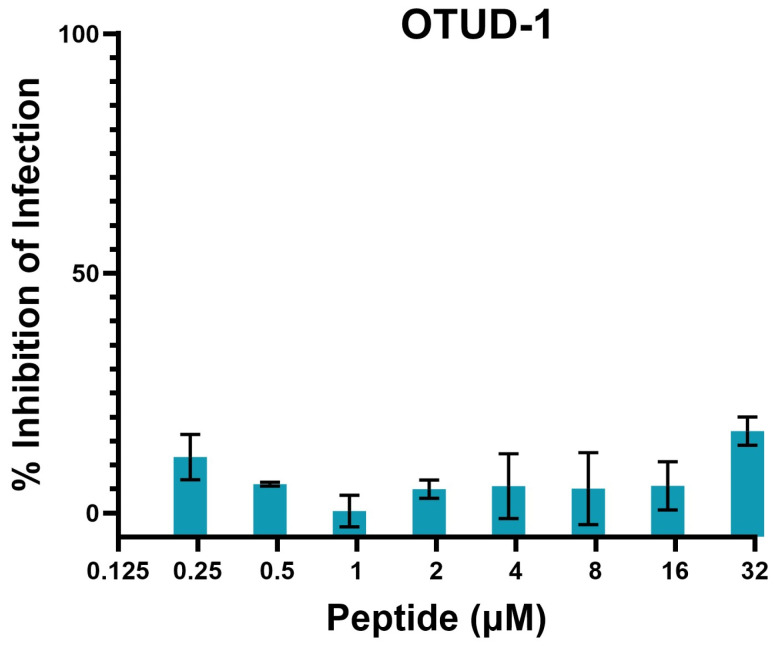
Inhibition of SARS-CoV-2 infection of Vero-E6/TMPRSS2 cells by OTUD1 peptide, attached to an octa-arginine cell-penetrating peptide (sequence RRRRRRRRGPDRNFRLSEHRQALA-am). Data is normalised against DMSO-treated infected cells. The plot shows the mean and standard deviation of 3 independent experiments.

Given that the OTUD1 peptide naturally occurs intracellularly, we assessed the antiviral effects of the OTUD1-derived peptide modified at the N-terminus with a cell-penetrating octa-arginine sequence. The sequence was then RRRRRRRRGPDRNFRLSEHRQALA-am, where “am” indicates amidation of the carboxy terminus. There was no evidence for any marked impact of this peptide on the inhibition of SARS-CoV-2 infection of Vero-E6/TMPRSS2 cells (Figure 5).

## 3. Discussion

Our interest in targeting the fusion peptide (FP) of SARS-CoV-2 spike (S) arose from its evolutionary sequence conservation relative to many other regions of the S protein, and its key role in viral entry by membrane fusion. Its choice as a target of interest was also guided by the success story of Enfuvirtide (Fuzeon)—a 36-residue-long biomimetic peptide modified from the fusion domain of HIV-type 1 virus that binds to the heptad repeat 1 (HR1) of the HIV-type 1 glycoprotein. The HR domain of the SARS-CoV-2 Spike protein undergoes a conformational shift from three helical bundles of HR1 and HR2 to a six helical bundle during viral-host membrane fusion [28]. Peptides derived from HR2 of SARS-CoV Spike have been reported to inhibit SARS-CoV infection [29]. While HR domain peptides targeting SARS-CoV-2 [5,30] are a useful strategy to develop anti-coronaviral peptides, the size of the currently effective peptides such as the pegylated cholesterol 46mer HR1-derived peptide pan-coronaviral inhibitor in phase II clinical trials [5] may place barriers to their cost-effective and easily delivered formulation, lowering their chance of progressing to widespread clinical use. While naturally generated antibodies can bind the initial segment of the FP region of a broad range of coronaviruses with high affinity [31], these are also large proteins.

In the interests of developing more tractable oral, intranasal or inhaled pancoronaviral therapeutics modelled on short peptides, we experimentally panned two libraries of phage displayed peptides. These were a combinatorial library of a billion 7-mer peptides and a proteomic phage display library of 16mer peptides whose sequences were derived from disordered regions of human proteins. To overcome the proteolytic sensitivity of L-peptide ligands to naturally occurring enzymes, we used a mirror-image phage display strategy in screening the L-peptide PhD7-mer library with a D-peptide FP bait. The ten lead peptides selected for in vitro screenings were then synthesised using D-amino acids, in order to avoid rapid proteolytic degradation in the cellular environment during in vitro screens. All ten D-peptides were non-toxic to the VeroE6/TMPRSS2 mammalian cells but had no anti-viral effects. We noted that the leading 7-mer peptides that bound FP did so less competitively than an artefactually out-competing false positive. This suggests that such small peptides may have a limited binding affinity. Thus, smaller canonical peptides may not be a good starting point for the design of FP-binding reagents.

Panning a library of larger 16mer peptides from a set of disordered regions of human proteins suggested a region of OTUD1, since the selection of two overlapping peptides is much less likely to reflect an artefactual enrichment. A peptide was synthesised based on their sequence, augmented by an N-terminal octa-arginine cell penetrating component, but this failed to inhibit viral infection in the cells. While the D-peptides synthesised from the previous PhD7 mirror phage display strategy were proteolytically resistant, the OTUD1 peptide could potentially have been inactivated prior to interaction with FP in the cell lines, which overexpress TPMRSS2 on their surface. TPMRSS2 can cleave after arginines and lysines, which are present in the octa-arginine-OTUD1 peptide tested (RRRRRRRRGPDRNFRLSEHRQALA-am). Thus, future FP-binding peptides designed to mimic OTUD1 may need to combine their enrichment for positive charges with proteolytic resistance.

OTUD1 is a de-ubiquitinase (DUB). At least 24 DUBs including OTUD1 negatively regulate virally induced antiviral IFN-I production, while another 10 DUBs positively regulate antiviral responses [32]. OTUD1 is one of 21 down-regulated signature genes associated with COVID induction of a long-term inflammatory T cell and NK cell states [33]. While it is possible that the OTUD1 peptide interaction with FP is mimicking the interaction of OTUD1 protein and FP during viral infection, it is also possible that the OTUD1 peptide may be mimicking some other natural ligand of FP. Either way, a better understanding of the peptide’s binding to FP could help in the design of more efficient FP inhibitors.

To explore the desirable properties of OTUD1-mimetic peptides or compounds, we carried out structural modelling. A three helix wedge shape conformation of FP has been observed on NMR analysis within membrane bicelles [6]. This conformation serves to expose hydrophobic residues on the surface where they can form interactions with the hydrophobic membrane components. Our Alphafold3 prediction of FP and OTUD1 peptide interaction indicated that the OTUD1 peptide bound a similar wedge-like conformation of FP in a stable interaction, with positively charged residues on one face of the OTUD1 peptide’s alpha helix making stable contacts with some hydrophobic residues in FP. Molecular dynamics modelling indicated that the interaction is overall stable, maintaining the interaction of positively charged OTUD1 residues, but the third FP helix re-oriented later in the simulation, suggesting alternative binding modes. The interfaces of antibodies against the N-terminal region of FP showed no such preponderance of positively charged residues [31], suggesting that the preference for positively charged residues in interaction with FP may come into play in the full FP structure stabilised by a disulphide bond. Of the four residues of FP that played consistent roles during MD simulation in salt bridge (Asp33) and hydrophobic (Leu6, Leu7 and Leu34) interactions with OTUD1, two are completely conserved in the fusion peptides of diverse coronaviruses [34], while two of the leucines are sometimes replaced by the similarly hydrophobic isoleucine or valine. This is promising for the design of pancoronaviral inhibitors focusing on these interacting residues. This modelling opens up the prospect of further computational and experimental screening of larger libraries of positively charged alpha helical or similar compounds for FP interaction.

In vitro assays of the identified peptides of most interest showed that none of them are strong inhibitors of viral infection. We hope that these negative results might stimulate more successful designs from other groups to lead to the development of fusion peptide modulating reagents with potential as pancoronaviral treatments. Such FP-targeting reagents might provide another tool in the arsenal of therapies for novel emerging coronavirus threats, of particular importance during the early stages in the emergence of novel coronaviral zoonotic epidemics before immunisation strategies can be put into place.

## 4. Materials and Methods

### 4.1. Peptide Synthesis and Characterisation

Peptides were synthesised as forward L-sequences, unless otherwise indicated. The phage display biotinylated FP bait and the peptides selected from phage display were synthesised with their L-amino acids substituted by D-amino acids. The side chain protecting groups were Boc for Trp, and Lys unless otherwise stated: tBu, for Ser, Thr, and Tyr; O-*t*Bu for Asp and Glu; Pbf, for Arg; Trt, for Asn, Cys, Gln and His. All the peptides were synthesised on a 0.1 mmol scale from a Rink Amide MBHA resin except for biotinylated fusion D-peptide FP where Rink Amide ProTide resin was used. Sequences were assembled by solid-phase peptide synthesis (SPPS), according to the Fmoc/t-Bu strategy, with DIC/Oxyma coupling chemistry, with DMF as a solvent, using a microwave-assisted Liberty Blue peptide synthesiser from CEM (CEM Microwave Technology Ltd., Dublin, Ireland). For coupling and deprotection steps, we followed the standard coupling with the standard deprotection step as recommended by CEM Liberty Blue peptide synthesiser manual. In brief, in the microwave-assisted Liberty Blue peptide synthesiser, the amino acid coupling steps by DIC/Oxyma were performed at 90 °C for approximately 100 s, and the deprotection steps with 20% piperidine were performed at 90 °C for approximately 1 min. After automated peptide assembly, modifications like biotinylation and acetylation were carried out manually before cleaving the peptide from resin, whereas peptide cyclization was carried out after cleaving the peptide from resin. For N-term acetylation, the Fmoc deprotected peptidyl resin was reacted with (1:1) acetic anhydride: DCM solution. The reaction was performed for 3 min initially and then repeated for 7 min. The resin was subsequently washed 3 times with DCM then dried.

The assembled peptides were released from the resin using a cleavage cocktail containing 81.5% TFA, 10% thioanisole, 5% water, 2.5% 1,2-ethane dithiol and 1% triisopropylsilane (TIS). The crude peptides were purified by reverse phase HPLC (Shimadzu Prominence LC system, Kyoto, Japan), using a C18 column (Gemini 110 Å, 5 μm, 10 × 250 mm, Phenomenex, Macclesfield, Cheshire, UK) over a gradient of water/acetonitrile with 0.1% TFA added to each buffer. The mass of the peptides was confirmed by MALDI TOF Mass Spectrometry (Bruker autoflex maX, Billerica, MA, USA).

The scheme for generating cyclic biotinylated FP fusion D-peptide is provided in Supplementary Figure S1. The peptide was assembled by automated SPPS, using a lysine at the C terminus with epsilon-amino protected by a Mtt group. The resin with N-terminally Fmoc-protected peptide was treated with a solution of 1% TFA, 2% TIS and 97% DCM to remove the Mtt group from the lysine side chain. Next, the resin with the free lysine side chain amine was reacted twice with 10 eq. of biotin, 10 eq. HATU and 20 eq. of diisopropylethyl amine (DIEA). The Fmoc deprotection of the N-terminus of the peptide was later carried out by adding 20% piperidine in DMF twice for 1 h each cycle to the resin. The peptide was then released from the resin using a cleavage cocktail as described above. After HPLC purification, the linear peptide was dissolved at a concentration lower than 100 µg/mL in 50 mM ammonium acetate, pH = 8, with continuous stirring under air for 24 h to form the heterodetic cyclic peptide [35].

### 4.2. Combinatorial NEB PhD Phage Display Screening

Streptavidin solution (Sigma S4762, Kawasaki, Japan, 1 mg/mL) (SA) was prepared in 1× PBS. A total of 9 mg of silica nanoparticles (SiO_2_ Nps 150 nm in ethanol) was washed with deionized H_2_O by spinning (12,000 g for 5 min at room temperature), and the COOH coating was activated to COO- by 0.2 M EDC and 50 mM NHS, then rinsed with dH_2_O and resuspended in 1× PBS. A measurement of 1 mL of SA was added to 9 mL of equilibrating buffer (10 mM NaOAc; pH 4.0). Then 1 mL of SiO_2_ Nps 150 nm (9 mg/mL) was added and rotated at room temperature for 4 h. The pellet of SA-conjugated nanoparticles was collected by spinning at 12,000 g for 5 min and washed twice by resuspension/rinsing in 1× PBS (900 µL). Finally, the pellet of SA-conjugated-Nps beads was resuspended in 1 mL of 1× PBS. Remaining 750 μL of SA-Conjugated-Nps beads were functionalized with 1 mL of D-FP12_biotin (0.5 mg/mL) on a rotating wheel for 30 min at room temperature. The functionalized beads were collected as a pellet by spinning at 12,000 g for 5 min and washed with 1× PBS twice. A final suspension of functionalized beads (1 mL 1× PBS) was stored at 4 °C. Biopanning experiments were performed in sterile microcentrifuge tubes (1.5 mL). After blocking 250 μL of functionalized beads with 750 μL of blocking buffer (5 mg/mL BSA in 1× PBS), 100 μL of Ph.D.-7 library (NEB #E8100S) was added and mixed on a rotating wheel for 30 min at room temperature. Unbound phage (target negative library) was separated from the phage-bound functionalized beads by spinning. The phage-bound functionalized beads collected as a pellet were washed and eluted with Glycine elution buffer (0.2 M Glycine-HCl, pH 2.2, 1 mg/mL BSA) and then neutralised with a neutralising buffer (1 M Tris-HCl, pH 9.1). The target-bound phage viruses were further amplified using *E. coli* (K12 ER2738) cells following the manufacturer’s protocol (Ph.D.-7 Phage display peptide library kit, NEB). The amplified target-bound phage viruses were screened against the target-free SA-Conjugated-Nps beads (250 μL) for target-negative screening to eliminate the peptides binding to SA and/or Nps. The target-positive phage viruses were further amplified and screened against functionalized beads for the second and third rounds of screening.

After the third round of biopanning, phage DNA was isolated using the QIAGEN (Venlo, The Netherlands) Plasmid Mini Kit (Cat No. 12123) and following the manufacturer’s protocol. The isolated phage DNA was PCR-amplified using the forward ATTCGCAATTCCTTTAGTGGTACC primer and the reverse CCCTCATAGTTAGCGTAACGATCT primer. The PCR product was purified using QIAquick (Venlo, The Netherlands) PCR purification kit and quantified using the NanoDrop (Wilmington, DE, USA) spectrophotometer and gel electrophoresis. The purified 177 bp PCR product sample was sent for sequencing (Illumina Novaseq sequencer, Eurofins Genomics, Ebersberg, Germany). The result files were processed using in-house Python scripts to rank the peptide sequences by copy number. The base tags used for 7-mer peptide search were “TATTCTCACTCT” for forward peptide sequences and “CGAACCTCCACC” for reverse complementary peptide sequences for reverse reads. The FASTQ files obtained had the non-tagged reads discarded. Reverse reads were reverse-complemented and merged with Forward reads. The merged dataset was translated to 7-mer peptides, preceded by Tyr Ser His Ser codons and followed by Gly Gly Gly Ser within the pIII coat of M13KE.

### 4.3. Surface Plasma Resonance

All SPR experiments were performed on Biacore T200, using Streptavidin SA sensor chip (Cytiva, Macquarie Park, NSW, Australia), immobilisation buffer (HBS-EP, 3 mM EDTA), and running buffer HBS-P (+/−2 mM Ca^2+^). Biotinylated peptides were dissolved in dH_2_O to 10 μg/mL and injected for 60 s to reach ~1/10 of Mr RU. The injection needle was cleaned with 1 M NaCl/50 mM NaOH between injections. Analyte peptides were dissolved in a running buffer and were injected at 1 micromolar concentration. The association phase was recorded for 500 s and the dissociation phase for a further 500 s. The running buffer was used to calibrate the assay to account for background noise. This baseline was used to calibrate the results shown. Plots of the sensorgrams were prepared using the R ggplot2 package.

### 4.4. Proteomic Phage Display

The selection experiment was performed in duplicate following the strategy previously described [21]. Thirty microliters of Streptavidin Dynabeads (Invitrogen, Waltham, MA, USA, 100099482) were taken in two different microcentrifuge tubes for spike-FP12 peptide and the control, respectively. Beads were washed with 700 µL of PBS buffer on the magnetic rack. The beads were blocked with 700 µL of PBS + 0.5% (*w*/*v*) BSA for 1 h at 4 °C. Beads for the control were washed three times with 700 µL of PT buffer (PBS + 0.05% Tween-20). HD2 näive phage library [21], with 10^10^ phages in 200 µL was prepared in PBS buffer. Phage library was added to the control beads and incubated at 4 °C for 1 h. Beads for spike-FP12 peptide were washed with 700 µL of PT buffer after 2 h of blocking. The unbound phage solution (200 µL) from the control beads was transferred onto beads for spike-FP12 peptide. Five micrograms of spike-FP12 peptide in 100 µL was also added onto the beads along with the phage solution to allow the binding. Beads were incubated for 2 h at 4 °C on the shaker. The spike-FP12 beads were washed five times with 700 µL of PT buffer. The bound phages were eluted with 100 µL of actively growing log-phase *E. coli*. Elution was performed at 37 °C, 200 rpm for 30 min on the shaker. The bacteria were hyper-infected with 10^11^ helper phages by incubating for 45 min at 37 °C, 200 rpm. Infected cells were transferred to 10 mL of 2YT medium supplemented with 30 µg/mL kanamycin, 100 µg/mL carbenicillin and 0.3 mM IPTG. Phages were amplified by incubating the cells overnight at 37 °C, 200 rpm. Next day, phages were harvested by centrifuging the tubes at 17,000× *g*, 4 °C, 10 min. The supernatant was transferred into fresh high-speed tubes. Phages were precipitated by adding 2.5 mL of PEG/NaCl buffer (20% PEG-8000 + 2.5 M NaCl) into the supernatant. The phages were incubated for 10 min on ice to precipitate. Tubes were centrifuged at 17,000× *g*, 4 °C for 10 min. The supernatant was discarded, and the pellet was suspended in 1 mL of PBS buffer. The phage solution was transferred into an autoclaved microcentrifuge tube and labelled as round 1 phages. Round 1 phages were used for another round of selection. The procedure was repeated for 3 rounds.

In order to identify the binding peptides, 5 µL of amplified phages from round 3 were PCR amplified. Phages were dual-barcoded using barcoding strategy [36], and the amplification was performed with Phusion High-Fidelity PCR Master mix (Thermo Scientific, Waltham, MA, USA). The size of the amplified product was confirmed on 2% agarose gel using 100 bp marker (Thermo Scientific). The PCR product was normalised with Mag-bind Total Pure NGS (Omega Bio-tek, Norcross, GA, USA) and eluted with 20 µL of TE (10 mM Tris-HCl, 1 mM EDTA, pH of 7.5) buffer. Eluted DNA was run on 2% agarose and purified using QIAquick Gel extraction Kit Qiagen kit. DNA was eluted with TE buffer, and the concentration was quantified with Quant-iT PicoGreen dsDNA Assay Kit (Molecular probes by Life technologies, Carlsbad, CA, USA). The NGS sample was sent to the NGS-NGI SciLifeLab facility (Solna) for sequencing and analysed with Illumina MiSeq v3 run, 1 × 150 bp read setup, 20% PhiX. Demultiplexing and data analysis were performed as described previously [21].

### 4.5. SARS-CoV-2 Infection Assay and Viability Assay Methods

All cell-based assays were performed using Vero-E6/TMPRSS2 cells (Cat #100978), obtained from Centre For AIDS Reagents (CFAR) at NIBSC [37,38]. Cells were maintained in Dulbecco’s Modified Eagle’ Medium (DMEM) (Gibco, Billings, MT, USA, Cat # 61965-026) supplemented with 10% Foetal Calf Serum (FCS, Gibco, Cat #: 10500-064), and 1 mg/mL geneticin (Gibco, Cat # 10131-035). Cells were incubated at 37 °C and 5% CO_2_ in a humidified incubator. Cells routinely tested negative for mycoplasma contamination.

Infection experiments were performed using ancestral SARS-CoV-2 isolate with D614G substitution (Pango lineage B.177.18, GenBank accession ON350866, Passage 4) [39]. Work was conducted in a Containment Level 3 laboratory under Biosafety Level 3 guidelines, using an existing high-throughput pipeline in place for drug screening and assessing viral neutralisation. Vero-E6/TMPRSS2 cells were seeded at 2.5 × 10^4^ cells per well in 96-well clear, flat-bottom plates and incubated overnight at 37 °C with 5% CO_2_ to reach 90–100% confluency at the time of infection. Cells were incubated with varying concentrations of test compounds or the corresponding vehicle control (PBS or DMSO) for 2 h prior to viral infection. Each compound was tested in a minimum of 2 independent experiments. Remdesivir (5 μM) was included as a positive control. Remdesivir acts as a nucleoside inhibitor, resulting in inhibition of RNA synthesis. Remdesivir inhibited infection by 95% (*n* = 10) (Supplementary Figure S5).

SARS-CoV-2 was then added at an MOI of 0.01 directly to each well. Plates were incubated at 37 °C with 5% CO_2_ for 18 h. Cells were infected with an MOI of 0.04 for 2 h, before being washed twice with dPBS and incubated in DMEM-2 containing fresh peptide or control for 18 h at 37 °C with 5% CO_2_. Cells were subsequently trypsinised (Trypsin-EDTA, Thermo Scientific, Cat #15400054) to obtain a single cell suspension and fixed in 4% formaldehyde (Sigma Aldrich, Darmstadt, Germany, F8775).

Flow cytometry staining was carried out as previously described [39]. Fixed cells were permeabilized in Perm/Wash solution (BD, Cat #51-2091KZ) for 30 min at room temperature, then incubated with an anti-SARS-CoV-2 nucleocapsid primary antibody (Invitrogen, Ref#: MA1-7403; RRID:AB_1018420) and an anti-mouse IgG fluorescein secondary antibody (Invitrogen, Cat #F2761; RRID:AB_2536524). Cells were resuspended in Phosphate Buffer Saline (PBS, Sigma, Cat #P4417) before flow analysis (Beckman Coulter CytoFlex, Indianapolis, IN, USA). Cells were gated with Forward and Side-Scatter gates to exclude debris from intact cells, with single cells identified using Area versus Height gating. Percentage SARS-CoV-2 nucleocapsid positive cells were detected using the FITC-channel (Blue 488 nm laser, 525/40 laser). Analysis was performed using CytExpert software (version 2.4.0.28, Beckman Coulter, Brea, CA, USA). The inhibition of infection was calculated as a percentage relative to the infected cells in the uninfected control wells and the respective vehicle control infected cells.

For the CCK-8 cell viability assay, cells were pre-treated with peptides as described above and mock-infected for 18 h at 37 °C with 5% CO_2_. Cell viability was measured using the cell counting kit-8 (CCK-8, Sigma, Cat #96992-3000TESTS-F) according to manufacturer’s instructions with a 2 h colour development before absorbance was measured at 450 nm (SpectraMax iD3).

### 4.6. Structural Modelling

OTUD1 peptide was initially investigated using Alphafold3 [24]. We then used the structural model of the OTUD1–FP complex generated with AlphaFold3 as starting coordinates for molecular dynamics (MD) simulations. The complex was subsequently processed and prepared for MD using CHARMM-GUI [40,41], following standard protocols for biomolecular systems. All-atom MD simulations were carried out with GROMACS [42] using the CHARMM36m [43] force field and the TIP3P water model for solvation. The solute (protein–peptide complex) was placed in a periodic cubic box with a minimal solute–box edge distance of 10 Å, solvated with explicit water and neutralised with counterions. Energy minimization was first performed for 1000 steps using the steepest descent algorithm to remove steric clashes. Subsequently, an equilibration protocol was applied: first under NVT ensemble (constant number of particles, volume and temperature) and then under NPT ensemble (constant pressure), to gradually relax the system. Hydrogen atoms were constrained using the LINCS algorithm. Long-range electrostatics were treated with the Particle Mesh Ewald (PME) method, and standard cutoffs (≈1.0–1.2 nm) were used for both Coulomb and van der Waals interactions, as implemented in the CHARMM36m force field. Production MD simulations were performed for 200 ns at 300 K with a 2 fs integration timestep, saving trajectory frames every 10 ps. Post-processing and trajectory analyses including root-mean-square deviation (RMSD), root-mean-square fluctuation (RMSF), minimum distances, contact analysis, and hydrogen-bond occupancy were performed using built-in GROMACS tools.

## 5. Conclusions

Combinatorial 7-mer phage display and proteomic phage display of peptides from human proteins identified potential candidate peptides binding FP, including a peptide derived from human OTUD1. However, none of these peptides had a marked impact on the in vitro infection of mammalian cells by SARS-CoV-2. The modelled structural binding mode of the OTUD1 peptide suggested a positively charged helical surface binding the hydrophobic surface of FP. This may give insights into the future design of peptides or other compounds targeting FP as pancoronaviral inhibitors.

## Figures and Tables

**Table 1:** Four spike-derived peptides screened for binding kinetics with FP by SPR.

Region of S	Residues	Sequence	Related Peptide
Heptad repeat	936–957 *	ac-DSLSSC(chol)ASALGKLADVVNQNAQ-am	HR [14]
Membrane embedding regions	788–806	IYKTPPIKDFGGFNFSQIL-am	WWI [15]
	891–906	GAALQIPFAMQMAYRF-am	IFP-WWII-R1[16]
	1095–1110	FVSNGTHWFVTQRNFY-am	IFP-WWII-R2 [16]

* with cholesterol at T941 > C replacement and with Q947 > K.

**Table 2:** Ten enriched PhD7-mer L-peptides tested in antiviral assays as D-peptides.

Sequence	Read Count	Occurrence in an Unrelated Screen [19]
WSLGYTG	6,280,489	4
NTWPYKP	264,257	1
HDTTLIT	169,773	-
SLTGTRF	11,170	-
RDHYMMF	7135	-
HFARHLA	7063	-
LTSLPRN	4284	-
WSRGYTG *	3676	-
LEVYYET	3570	-
ETSTMYP	3552	-

* Likely mutational variant of the artefactually over-represented WSLGYTG.

**Table 3:** Read counts of human disordered protein region phage display binding to FP.

Gene Name	Protein Name	Peptide	Count	% Count
*CFAP73*	Cilia- and flagella-associated protein 73	QLEHVKLFMQDLSAML	4673	9.07
*OTUD1*	OTU domain-containing protein 1	GPDRNFRLSEHRQALA	3393	6.59
*CFAP65*	Cilia- and flagella-associated protein 65	DTLLPTQQAEVLHPVV	1392	2.70
*DBP*	D site-binding protein	TLPFGDVEYVDLDAFL	148	0.29
*ACACB*	Acetyl-CoA carboxylase 2	GSSYAEMEVMKMIMTL	31	0.06
*MAP1A*	Microtubule-associated protein 1A	SFQYADIYEQMMLTGL	5	0.01
*MBTPS1*	Membrane-bound transcription factor site-1 protease	HPNIKRVTPQRKVFRS	3	0.01
*OTUD1*	OTU domain-containing protein 1	NFRLSEHRQALAAAKH	2	0.00

## Data Availability

The peptide sequences identified in the phage display experiment are submitted to the Xenodo repository DOI: 10.5281/zenodo.17898744.

## Supplementary Materials

The following supporting information can be downloaded at: https://www.mdpi.com/article/10.3390/molecules31020282/s1.

## Abbreviations

The following abbreviations are used in this manuscript:

FP	Fusion Peptide
SPR	Surface Plasmon Resonance
PCR	Polymerase Chain Reaction
-am	Amidation
ac-	Acetylation
NMR	Nuclear Magnetic Resonance
PDB	Protein Database
S	Spike Protein
nsp3	Non-structural protein 3
DUB	Deubiquitinase
OTUD1	Ovarian Tumour Deubiquitinase 1
MD	Molecular Dynamics
