# Supplementary material for: Screening for Peptides to Bind and Functionally Inhibit SARS-CoV-2 Fusion Peptide Using Mirrored Combinatorial Phage Display and Human Proteomic Phage Display
Source: Molecules. 2026 Jan 13;31(2):282. doi: 10.3390/molecules31020282 (PMC12844208; doi:10.3390/molecules31020282)
Supplement: Supplementary file 1 [file molecules-31-00282-s001.zip › molecules-3995626-supplementary.pdf]

Supplementary Materials

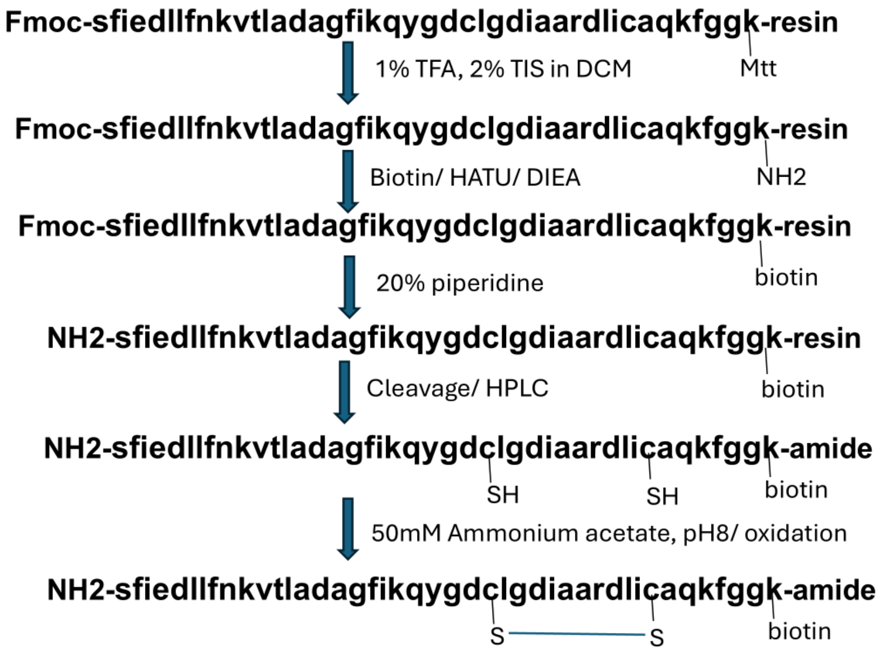

Supplementary Figure S1. Fusion peptide synthesis.

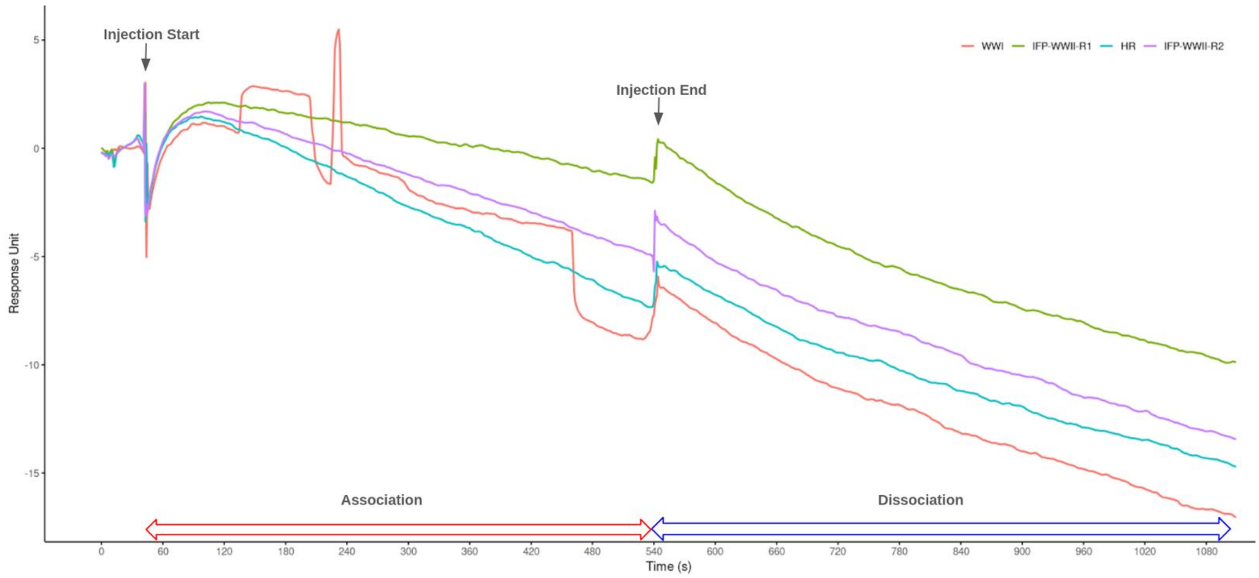

Supplementary Figure S2. Sensorgrams of HR, WWI, IFP-WWII-R1 and IFP-WWII-R2 binding to FP immobilized on Channel 2 (F2) of Streptavidin SA sensor chip. Analytical conditions: HBS-P running buffer; injection time of 500 seconds. Sensorgrams were obtained after reference cell subtraction (F2-1). Channel 1 (F1) was left empty and used as the reference flow cell.

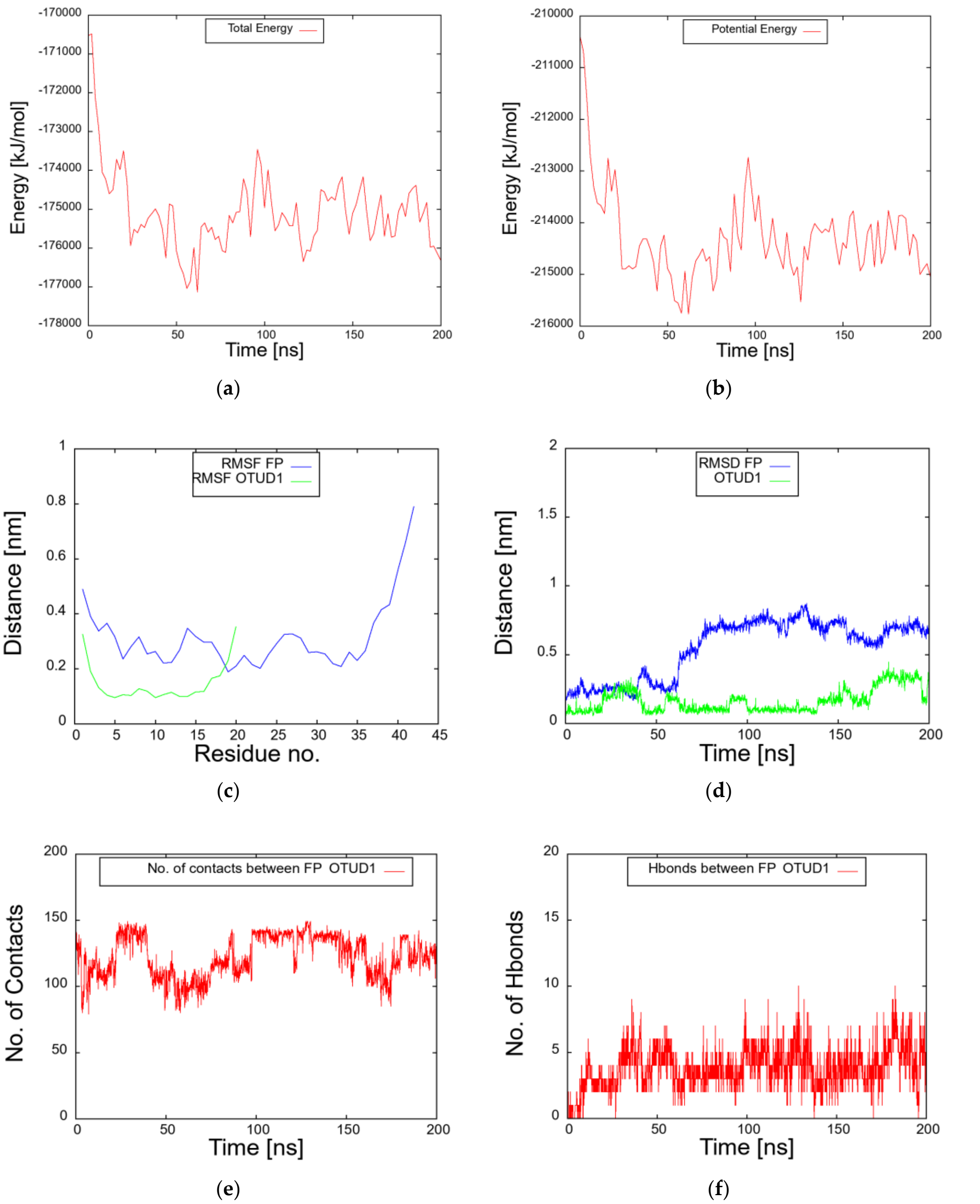

Supplementary Figure S3. Analysis of MD trajectories during 200ns simulations (a) Total energy of the system (b) Potential energy of the system (c) RMSF of OTUD1 and FP (d) backbone RMSD of OTUD1 and FP (e) no. of contacts per frame between FP and residues (4, 7, 8,11, 12, 15, 19) of OTUD1 (f) no. of hydrogens bonds between FP and OTUD1 over 200ns simulation.

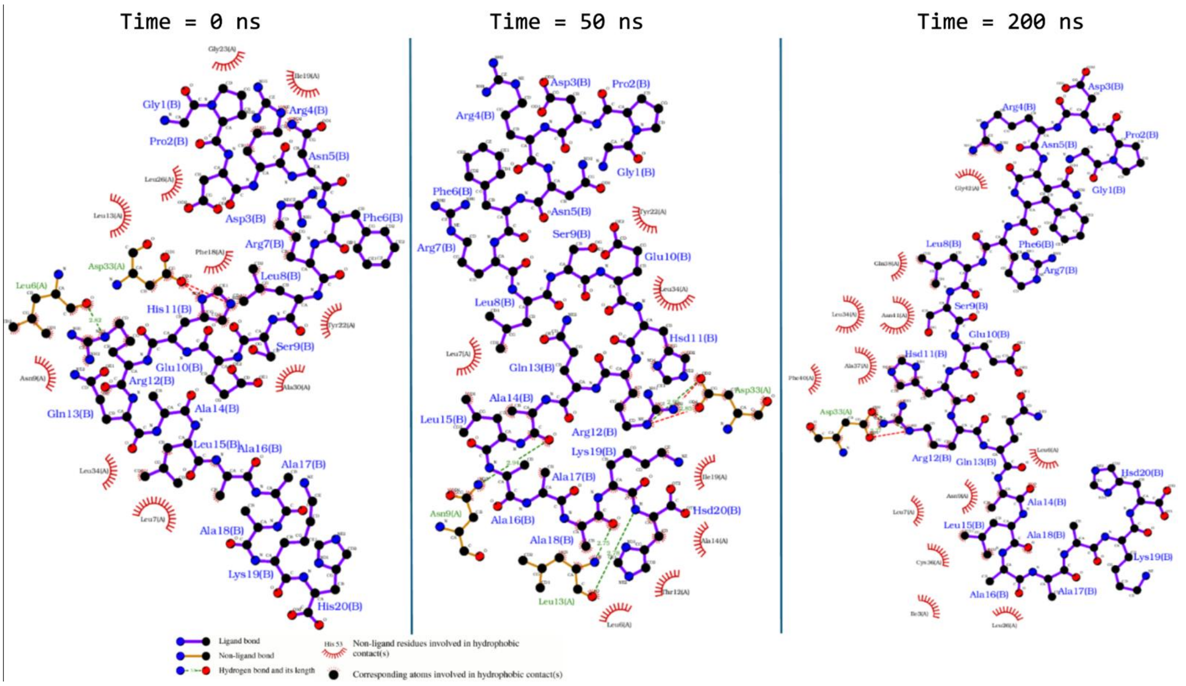

Supplementary Figure S4. LIGPLOT+ visualisation of key hydrophobic, hydrogen bond, and salt bridge interactions at 0, 50 and 200 ns of MD modelling. Salt bridges are denoted by a dashed red line, hydrogen bonds by a dashed green line, and hydrophobic interactions among residues by radiating red lines oriented towards the residues of interaction. The entire non-hydrogen structure of OTUD1 peptide is shown, with the atomic detail only given for FP residues involved in hydrogen bonds or salt bridges.

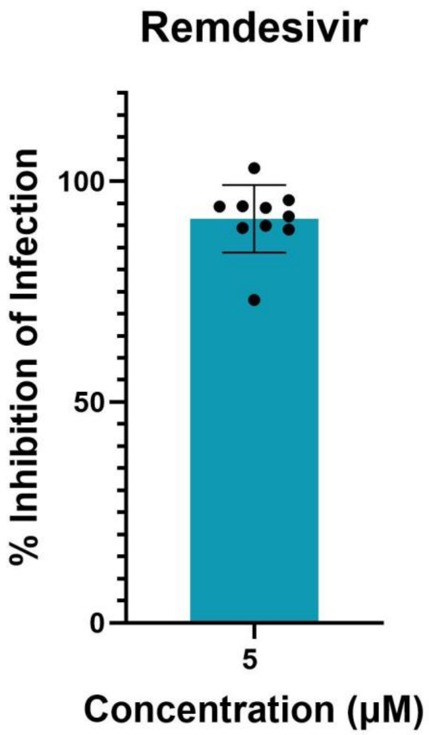

Supplementary Figure S5. Inhibition of SARS-CoV-2 Infection of Vero-E6/TMPRSS2 cells by Remdesivir (5µM). Data is normalised against DMSO-treated infected cells. Plot shows the mean and standard deviation of 10 independent experiments.
